# Draft Genome Sequences of *Streptococcus agalactiae* Strains Isolated from Nile Tilapia (*Oreochromis niloticus*) Farms in Thailand

**DOI:** 10.1128/genomeA.01300-14

**Published:** 2014-12-11

**Authors:** Pattanapon Kayansamruaj, Nopadon Pirarat, Hidehiro Kondo, Ikuo Hirono, Channarong Rodkhum

**Affiliations:** a Department of Veterinary Microbiology, Faculty of Veterinary Science, Chulalongkorn University, Bangkok, Thailand; b Department of Veterinary Pathology, Faculty of Veterinary Science, Chulalongkorn University, Bangkok, Thailand; c Laboratory of Genome Science, Tokyo University of Marine Science and Technology, Tokyo, Japan

## Abstract

During 2009–2011, two clinical and one environmental strains of *Streptococcus agalactiae* were isolated from Nile tilapia (*Oreochromis niloticus*) farms in Thailand. Draft genome sequences of two clinical isolates comprise 2,048,343 and 2,105,006 bp, while environmental isolates comprise 2,097,115 bp, having 1,573 to 1,578 coding sequences, respectively.

## GENOME ANNOUNCEMENT

*Streptococcus agalactiae* is the Gram-positive bacterial pathogen that produces serious disease in several mammalian and aquatic animal species (1–3). Several marine and fresh water fishes, including Nile tilapia (*Oreochromis niloticus*), have been reported to be susceptible to *S. agalactiae* infection, causing tremendous economic loss (1, 4). To date, the complete genome sequences of several *S. agalactiae* strains recovered from different countries have been investigated and can be found in the GenBank database. However, the genomic-scaled information of the *S. agalactiae* obtained from the Southeast Asia region is still unavailable. Therefore, the genome sequences of *S. agalactiae* isolated from Nile tilapia farms in Thailand were determined in this study.

Two strains of *S. agalactiae* (FNA07 and FPrA02) were collected from diseased Nile tilapia in 2009 and 2010, while another strain (ENC06) was recovered from water collected from an earthen tilapia pond in 2011. The genome sequencing process was carried out using the Illumina MiSeq platform with 251 paired-end run cycles (Illumina, USA). Once the sequencing process was accomplished, paired-end reads of poor quality were filtered out using the CLC Genomic Workbench version 6.9 (CLC bio, USA) and *de novo* assembly was conducted later. A total of 80 to 106 contigs with 25× average coverage were obtained after the assembly process. The order and orientation of contigs were determined by aligning to *S. agalactiae* strain GD201008-001 (accession number CP003810) using the Contiguator version 2.0 web-based service (5). Draft genome sequences of FNA07, FPrA02, and ENC06 contain 2,105,006, 2,048,343, and 2,097,115 bp, with G+C contents of 35.4, 35.4, and 35.5%, respectively. RNAmmer (6) analysis revealed the number of copies of 5S (*n* = 3; except for FPrA02, *n* = 2), 16S (*n* = 1), and 23S rRNA (*n* = 1) contained in the *S. agalactiae* genomes. Annotation of the genome assemblies was performed using RAST version 4.0 (7), which predicted 2,090, 2,032, and 2,083 coding sequences (CDS). In total, the coding genes were categorized into 341–343 subsystems. Among the CDSs, 19% were responsible for carbohydrate metabolism, 11% for protein metabolism, 9% for cell wall and capsule synthesis, 9% for amino acids and derivatives synthesis, 7% for RNA metabolism, 6% for DNA metabolism, 6% for nucleosides and nucleotides synthesis, and 6% for cofactors, vitamins, prosthetic groups, and pigments. Assigning the proteomes obtained by the RAST server to the OrthoMCL web service (8), about 1,383–1,393 orthologous groups were predicted and approximately 86% of them (1,203–1,205 groups) share common orthologs with other *S. agalactiae* reference strains (accession numbers AE009948, CP000114, CP003810, CP003919, AL732656). The MLST version 1.7 web tool (9) indicated that all 3 strains included in the current study belong to sequence type 7. Prophage identification using the PHAST search tool (10) showed 2 prophage regions (31 and 16 kbp in size) in the FPrA02 and ENC06 genomes, while 3 prophage regions (31, 16 and 8 kbp) were predicted in the FNA07 genome.

### Nucleotide sequence accession numbers.

The draft genome sequences of *S. agalactiae* strains FNA07, FPrA02, and ENC06 have been deposited at GenBank under the accession numbers JMBI00000000, JMBJ00000000, and JMBK00000000, respectively. The BioProject identification number is PRJNA244605.
